# Electron-Impact Total Ionization Cross Sections of CH and C_2_H_2_

**DOI:** 10.6028/jres.102.046

**Published:** 1997

**Authors:** Yong-Ki Kim, M. Asgar Ali, M. Eugene Rudd

**Affiliations:** National Institute of Standards and Technology, Gaithersburg, MD 20899-0001; Department of Chemistry, Howard University, Washington, DC 20059; Department of Physics and Astronomy, University of Nebraska-Lincoln, Lincoln, NE 68588-0111

**Keywords:** acetylene, hydrocarbon, radical, total ionization cross section

## Abstract

Electron-impact total ionization cross sections for the CH radical and C_2_H_2_ (acetylene) have been calculated using the Binary-Encounter-Bethe (BEB) model. The BEB model combines the Mott cross section and the asymptotic form of the Bethe theory, and has been shown to generate reliable ionization cross sections for a large variety of molecules. The BEB cross sections for CH and C_2_H_2_ are in good agreement with the available experimental data from ionization thresholds to hundreds of eV in incident energies.

## 1. Introduction

The Binary-Encounter-Bethe (BEB) model [1] has been successfully used to calculate the total ionization cross sections of a wide range of molecules [2–4] including H_2_, O_3_, and SF_6_. The BEB model combines a modified form of the Mott cross section with the asymptotic form of the Bethe theory (i.e., high incident energy) for electron-impact ionization of an atom.

These two theories are combined in such a way that not only the ionization cross section has the proper asymptotic form but so does the stopping cross section, which is the product of the cross section and the energy transfer from the incident particle to the target [1]. Although the theoretical model in its differential form requires the knowledge of continuum dipole oscillator strengths d*f*/d*E*, we assumed a simple form for d*f*/d*E* to facilitate the integration over the ejected electron energy. The resulting expression for the ionization cross section per molecular orbital, which we refer to as the BEB model, has a remarkably simple form [1]:

$$\sigma_{\text{BEB}}=\frac{S}{t+u+1}\left[\frac{\ln t}{2}\left(1-\frac{1}{t^{2}}\right)+1-\frac{1}{t}-\frac{\ln t}{t+1}\right],$$ (1)

where *t* = *T*/*B*, *u* = *U*/*B*, *S* = 4π*a*_0_^2^*NR*^2^/*B*^2^, *a*_0_ is the Bohr radius (= 0.5292 Å), *R* is the rydberg energy (= 13.6057 eV), *T* is the incident electron energy, and *N*, *B*, and *U* are the electron occupation number, the binding energy, and the average kinetic energy of the orbital, respectively.

In this article, we compare the BEB cross sections for the CH radical in its ground state ^2^P and C_2_H_2_ also in the ground state ^1^Ʃ_g_^+^ with available experimental data. Both molecules are used in modeling the behavior of edge plasmas in the divertor region of a tokamak [5].

## 2. Results and Discussion

The molecular constants *B* and *U* were calculated using the 6-311+G(d,p) basis set in the GAMESS code [6]. Although one can in principle use theoretical values for all *B*’s, theoretical values for the lowest ionization potential (IP) are usually inaccurate. Therefore, we used experimental values [7] only for the lowest IP to match the observed thresholds. Theoretical results and comparisons to experimental data presented below are also available on a NIST web page for electron-impact ionization cross sections at:

http://physics.nist.gov/PhysRefData/Ionization/Xsection.html
Note that the above address is case sensitive.

### 2.1 CH (^2^II)

**Fig. 1:**
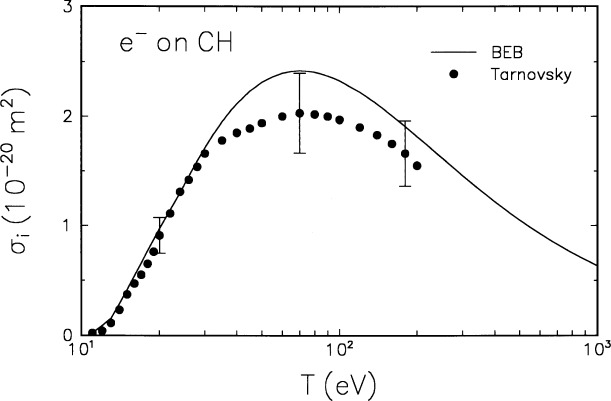
Comparison of the BEB cross section to experiment for CH. Solid curve, BEB cross section; circles, experimental data by Tarnovsky et al. (for CD) [8]. The ordinate is the ionization cross section, and the abscissa is the incident energy.

The molecular orbital constants *B*, *U*, and *N* for the CH radical are listed in Table 1, and the BEB model is compared in Fig. 1 to the only available experimental data by Tarnovsky et al. [8]. Although the experiment was performed using deuterium instead of hydrogen, i.e., with CD, our theory is insensitive to the use of the isotope. As Tarnovsky et al. [8] have pointed out, their experiment did not detect fast deuterons produced, which may partly account for the difference between theory and experiment near the peak. Otherwise, theory and experiment are in excellent agreement, particularly for *T* < 30 eV. This is the region of *T* for which most other theories have difficulty in reproducing observed cross sections. The BEB model produces reliable cross sections for many molecules at low *T* (≈ 50 eV or less) [2–4].

### 2.2 Acetylene, C_2_H_2_ (^1^Ʃ_g_^+^)

**Fig. 2:**
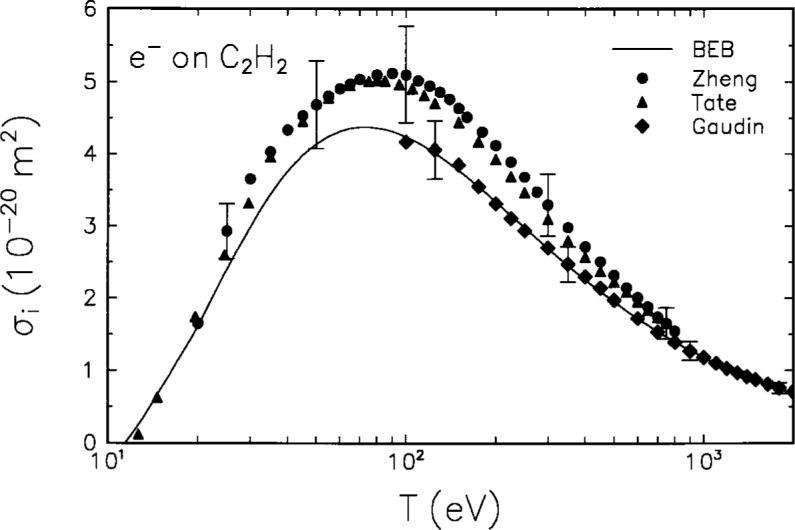
Comparison of BEB cross section to experiments for C_2_H_2_. Solid curve, BEB cross section; circles, experimental data by Zheng and Srivastava [9]; triangles, data by Tate and Smith [10]; diamonds, data by Gaudin and Hagemann [11]. The ordinate is the ionization cross section, and the abscissa is the incident energy.

The molecular orbital constants for acetylene are listed in Table 2, and the BEB model is compared in Fig. 2 to the available experimental data [9–11]. The experimental data by Zheng and Srivastava [9] and those by Tate and Smith [10] agree well with each other but they are approximately 15 % higher than the theory at the peak. On the other hand, the data by Gaudin and Hagemann [11] are in excellent agreement with the theory from *T* = 100 eV to 2 keV. Since the BEB model provides a convenient analytic form for the ionization cross section for the entire range of *T* shown in Fig. 2, and the data by Tate and Smith agree well with the theory for *T* < 30 eV, we are confident that the BEB cross sections will be appropriate and useful for plasma modeling applications.

## Figures and Tables

**Table 1:** Molecular orbital (MO) constants for CH (^2^II): *B* = binding energy, *U* = kinetic energy, *N* = occupation number. The *B* value marked by ^*^ is an experimental value

MO	*B* (eV)	*U* (eV)	*N*
1σ	309.23	436.80	2
2σ	23.48	36.40	2
3σ	13.03	32.96	2
1π	10.64^*^	26.19	1

**Table 2:** Molecular orbital (MO) constants for C_2_H_2_ (^1^Ʃ_g_^+^): *B* = binding energy, *U* = kinetic energy, *N* = occupation number. The *B* value marked by ^*^ is an experimental value

MO	*B* (eV)	*U* (eV)	*N*
1σ_g_	305.62	435.15	2
1σ_u_	305.50	436.31	2
2σ_g_	28.18	49.60	2
2σ_u_	20.80	32.79	2
3σ_g_	18.55	33.64	2
1π_u_	11.4^*^	28.99	4
